# A Case of Bronchial Dieulafoy's Disease Presenting as an Endobronchial Polypoid Lesion That Regressed After Bronchial Artery Embolisation

**DOI:** 10.1002/rcr2.70450

**Published:** 2026-01-05

**Authors:** Masayoshi Higashiguchi, Yu Yamaguchi, Satoshi Tetsumoto, Hideo Ishikawa

**Affiliations:** ^1^ Department of Respiratory Medicine and Clinical Immunology Suita Municipal Hospital Osaka Japan; ^2^ Hemoptysis and Pulmonary‐Circulation Center, Eishinkai Kishiwada Rehabilitation Hospital Osaka Japan

**Keywords:** bronchial artery embolisation, bronchial Dieulafoy's disease, hemoptysis

## Abstract

A 37‐year‐old woman presented to the hospital with a history of a small amount of hemoptysis. Bronchoscopy revealed a polypoid lesion in the left main bronchus. Due to oozing bleeding from the lesion, biopsy was deferred to avoid fatal haemorrhage risk. Bronchial angiography revealed an abnormal tortuous branch with pulmonary artery shunting. Bronchial artery embolisation with coils was successfully performed without complications. Bronchial Dieulafoy's disease was diagnosed. Follow‐up bronchoscopy approximately 4 months post‐embolisation showed regression of the polypoid lesion, with residual scar formation. Subsequently, the hemoptysis resolved completely. Dieulafoy's disease is characterised by a large‐calibre, submucosal artery lying just beneath the mucosa, predisposing to abrupt haemorrhage despite minimal or no mucosal defect. Although this disease has been classically described in the gastrointestinal tract, analogous lesions also occur in the bronchial tree. This case highlights the visual confirmation of lesion regression by bronchoscopy after bronchial artery embolisation.

## Graphical abstract

Bronchial Dieulafoy's disease is extremely rare and characterised by a large‐calibre, submucosal artery lying just beneath the mucosa, predisposing to abrupt haemorrhage despite minimal or no mucosal defect. This case highlights the visual confirmation of lesion regression by bronchoscopy after bronchial artery embolisation.

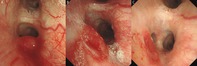

## 1 Introduction

Dieulafoy's disease is characterised by a large‐calibre, submucosal artery lying just beneath the mucosa, predisposing to abrupt haemorrhage despite minimal or no mucosal defect. Although this disease has been classically described in the gastrointestinal tract, analogous lesions also occur in the bronchial tree [1, 2, 3]. Bronchial Dieulafoy's disease is extremely rare. Because it mimics a neoplastic disease in bronchoscopic findings, it should be noted that biopsy of the lesion may lead to life‐threatening bleeding [1]. Here, we report the case of a patient with bronchial Dieulafoy's disease, who was treated by bronchial artery embolisation (BAE), with serial documentation of post‐embolisation regression using follow‐up bronchoscopy and contrast‐enhanced CT, complemented by convex‐probe EBUS confirmation of submucosal arterial flow.

## 2 Case Report

A 37‐year‐old woman presented to a clinic with a few days' history of a small amount of hemoptysis. Chest computed tomography (CT) revealed an endobronchial nodular lesion in the left main bronchus. Four days later, the patient was referred to the hospital for further evaluation. Upon referral, she appeared healthy and in no respiratory distress with an oxygen saturation of 98% while breathing ambient air. Her medical history included pneumonia treated with antibiotics at age 12 and hypothyroidism diagnosed at age 26, managed with levothyroxine. At the time of referral, she was not on any regular medication and had never smoked.

**FIGURE 1:**
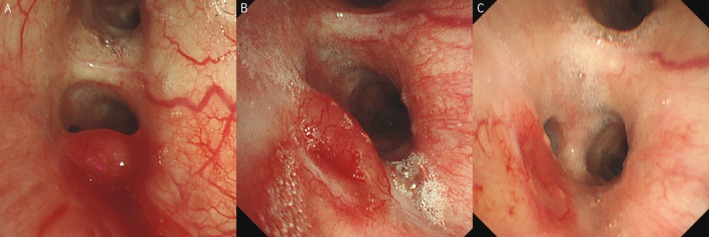
Bronchoscopic findings. (A) Polypoid lesion in the left main bronchus slightly proximal to the second carina was observed. (B) Polypoid lesion had regressed at 1 month post‐embolisation. (C) Polypoid lesion had disappeared with residual scar formation at 4 months post‐embolisation.

**FIGURE 2:**
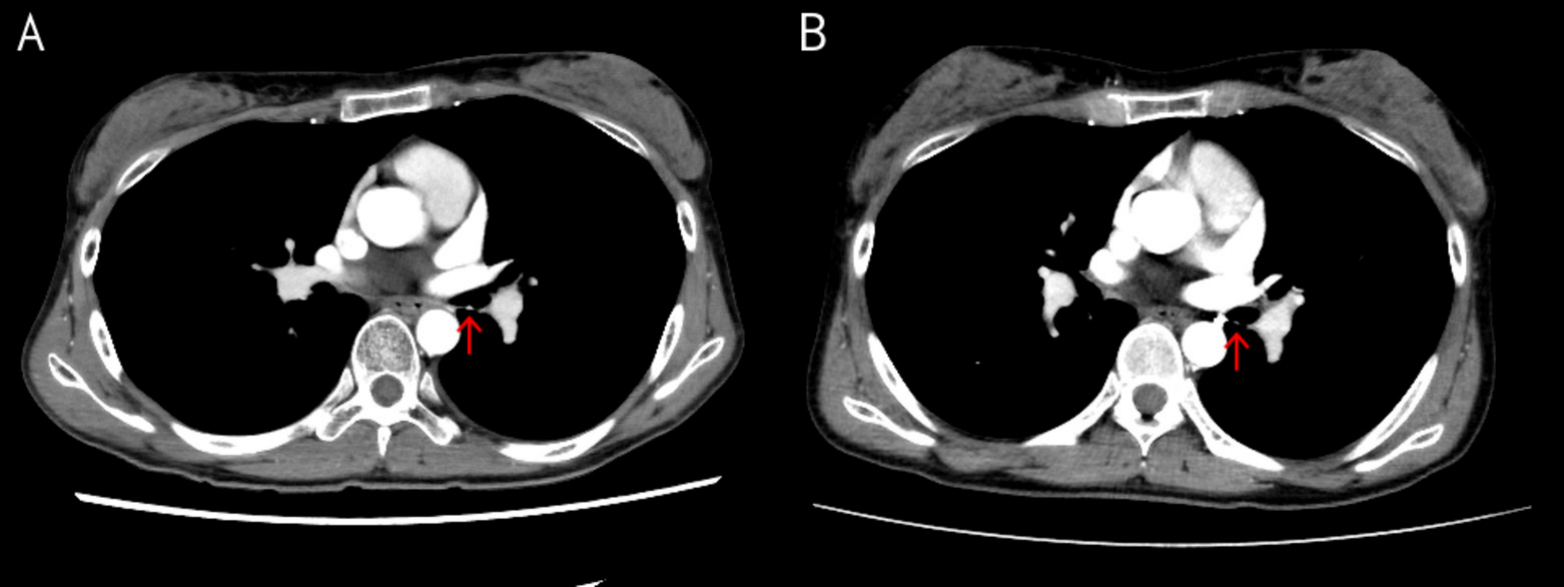
Contrast‐enhanced CT. (A) Endobronchial nodular lesion was enhanced by contrast material. (B) Endobronchial nodular lesion regressed with decreased enhancement after the bronchial artery embolisation.

**FIGURE 3:**
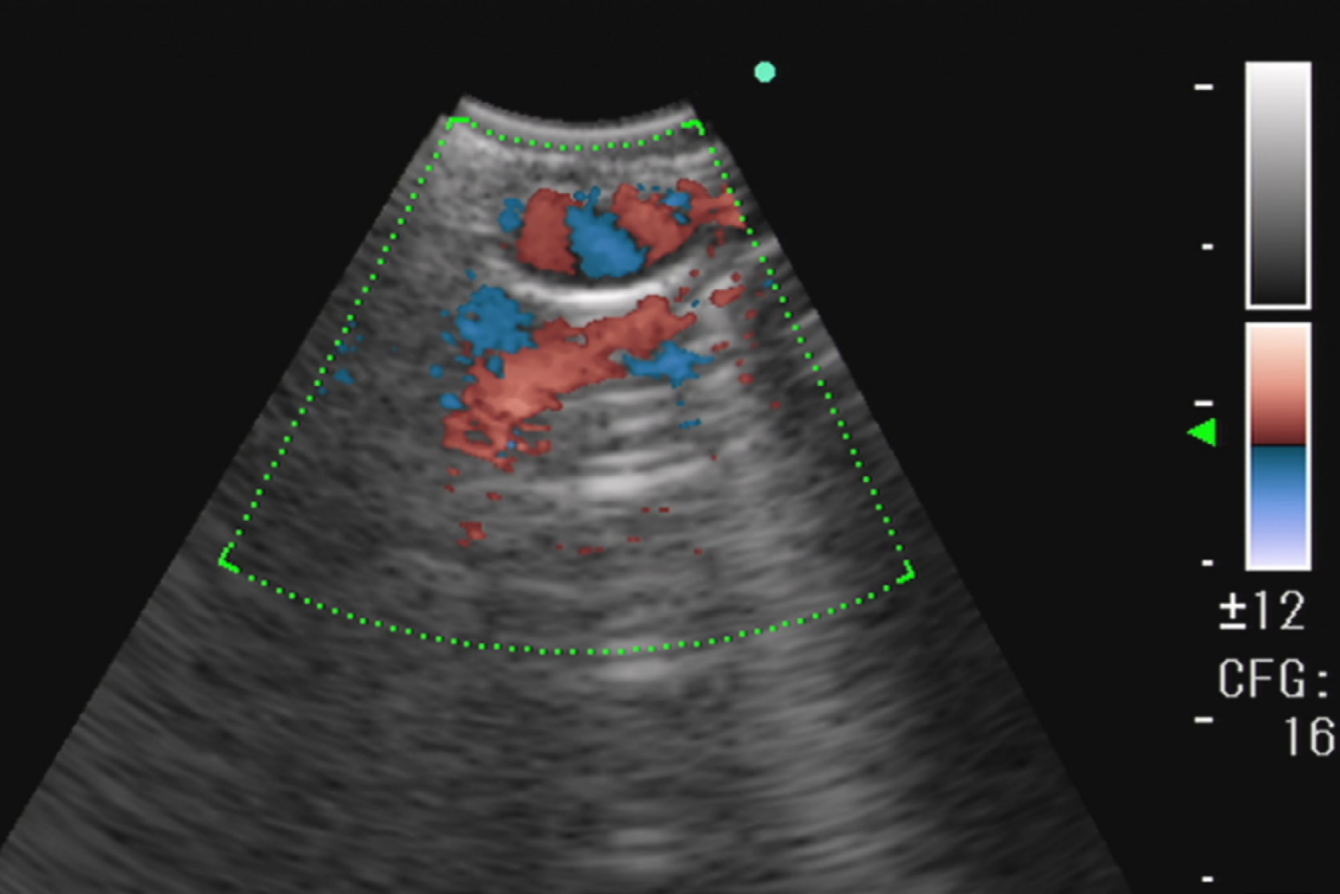
Convex‐probe endobronchial ultrasound. Abnormal blood flow beneath the polypoid lesion was seen.

Bronchoscopy revealed a polypoid lesion in the left main bronchus slightly proximal to the second carina (Figure 1A). Due to oozing bleeding from the lesion, biopsy was deferred to avoid fatal haemorrhage risk. Contrast‐enhanced chest CT demonstrated lesion enhancement, confirming the potential for life‐threatening haemorrhage with biopsy (Figure 2A). The CT imaging demonstrated a single left bronchial artery probably feeding the bronchial lesion and did not identify involvement of any other vasculature, including non‐bronchial systemic arteries. No abnormal opacities were observed in the lungs. Repeat bronchoscopy with convex‐probe endobronchial ultrasound (EBUS) revealed abnormal blood flow beneath the polypoid lesion (Figure 3).

**FIGURE 4:**
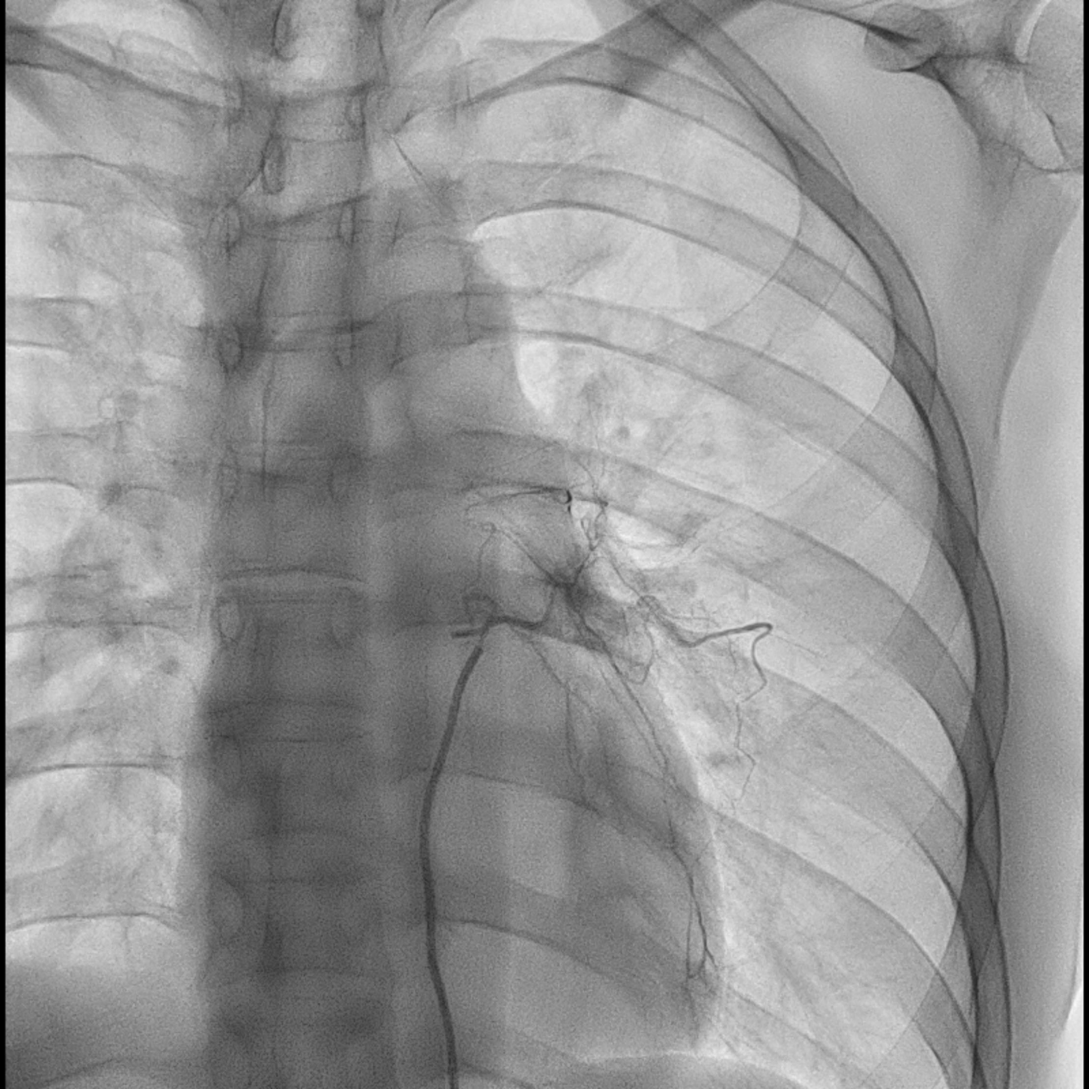
Bronchial angiography. Abnormal tortuous branch originating from the aorta at the level of the seventh thoracic vertebra with pulmonary artery shunting was seen.

She was then transferred to a specialised center for BAE. Bronchial angiography revealed an abnormal tortuous branch with pulmonary artery shunting (Figure 4). The 1.9–2.9 Fr microcatheter (Breakthrough, Boston Scientific Japan, Tokyo, Japan) was advanced into the left bronchial artery originating from the descending aorta at the level of the seventh thoracic vertebra. BAE was performed using AZUR Soft 3D coils (Terumo, Tokyo, Japan) (1.0 mm × 3 cm, *n* = 4; 1.5 mm × 4 cm, *n* = 3), achieving complete obstruction of the culprit artery without complications. The patient radiation exposure was 112.02 mGy. A total amount of 25 mL of 61.24% iopamidol was administered as a contrast agent. The total procedure time was 67 min. Based on the clinical, bronchoscopic and angiographic findings, bronchial Dieulafoy's disease was diagnosed.

The patient experienced intermittent episodes of small‐volume hemoptysis that appeared self‐limited, although oral hemostatic agents were prescribed. Follow‐up bronchoscopy showed that the polypoid lesion had regressed at 1 month post‐embolisation (Figure 1B), and had disappeared with residual scar formation at 4 months post‐embolisation (Figure 1C). Subsequently, the hemoptysis resolved completely. Consistently, contrast‐enhanced chest CT showed regression and decreased enhancement of the endobronchial nodular lesion (Figure 2B).

At 12‐month follow‐up, no recurrent hemoptysis or procedure‐related adverse events had been observed.

## 3 Discussion

The present patient presented with hemoptysis, and an endobronchial lesion was detected on chest CT. Bronchoscopic biopsy was deferred because oozing bleeding from the lesion was observed. The patient was diagnosed with bronchial Dieulafoy's disease based on bronchial angiography and was treated with BAE. Lesion regression was confirmed by bronchoscopy. This case adds incremental value to the existing literature in that post‐embolisation regression of an endobronchial polypoid lesion is serially documented by paired follow‐up bronchoscopy and contrast‐enhanced CT, and submucosal arterial flow is demonstrated using convex‐probe EBUS Doppler, supporting a vascular aetiology and helping avoid hazardous biopsy. Compared with prior reports (e.g., Qian et al. [1] and Chen et al. [4]), the multimodal documentation before and after BAE could provide practical value for diagnosis and follow‐up.

Bronchial Dieulafoy's disease can be diagnosed based on bronchoscopic findings, bronchial angiography, and histopathological examination. Bronchoscopic findings have been described as nodular, protruding, or polypoid [1]. The lesion can mimic an endobronchial tumour, but obtaining a biopsy for diagnosis is dangerous. Qian et al. analysed 73 patients with bronchial Dieulafoy's disease identified by literature search, and found that 6 of 19 patients who underwent bronchoscopic biopsy died due to ensuing massive haemorrhage [1]. Moreover, bronchoscopic biopsy was not diagnostic in most of the cases [1]. Convex‐probe EBUS can detect abnormal blood flow in the submucosa, which can aid diagnosis [1, 3, 5]. Bronchial angiography can reveal a dilated tortuous bronchial artery branch feeding the bronchial lesion and shunting with the pulmonary artery [1, 3, 4, 5]. Histopathological findings, which have usually been reported using surgical specimens, include thick‐walled abnormal blood vessels in the submucosa that can connect to the bronchial lumen [2, 3, 5].

Other types of endobronchial polypoid lesions with vascularity (e.g., carcinoid tumour, endobronchial arteriovenous malformation, hypertrophied bronchial artery, broncholith with reactive granulation, and endobronchial hemangioma) should be considered in the differential diagnosis. Given the risk of catastrophic haemorrhage, bronchoscopic biopsy should be deferred when the lesion is suspected to be associated with vasculature, and vascular evaluation using contrast‐enhanced CT and Doppler assessment with convex‐probe EBUS (and bronchial angiography when indicated) should be prioritised. Notably, several reported cases of bronchial Dieulafoy's disease lack pulsatile appearance on bronchoscopy [3, 4, 5]; therefore, the absence of visible pulsation does not exclude this diagnosis. In our case, conspicuous enhancement of the lesion on contrast‐enhanced CT and abnormal blood flow beneath the lesion on Doppler assessment were particularly helpful. These findings supported a vascular aetiology such as bronchial Dieulafoy's disease and reinforced the decision to avoid biopsy and proceed with further vascular evaluation.

Although the optimal management of bronchial Dieulafoy's disease has not been established, BAE is generally considered a preferred initial approach given its less invasive nature compared with surgery [1, 4, 5, 6]. Multiple embolisation procedures may be required in some patients, and surgical resection of the affected lobe is typically reserved for cases in which BAE fails [1, 3, 5]. Because the natural history and prognosis of the disease remain to be clarified, it is unknown whether selected patients with mild symptoms can be safely managed with watchful waiting. Notably, robust original data defining recurrence rates specifically after BAE for bronchial Dieulafoy's disease are lacking. However, the mechanisms of rebleeding after BAE (e.g., recanalisation or collateral recruitment) [6] are not unique to this condition and are also observed in hemoptysis of other etiologies [6]; therefore, contemporary outcomes of BAE for hemoptysis may provide a reasonable surrogate. With recent technical advances, BAE achieves approximately 90% haemostasis at 2 years [6, 7]. In addition, embolisation outcomes for hemoptysis using NBCA or PVA are reported to be broadly comparable [8], supporting the expectation that superselective coil embolisation can provide sufficiently durable haemostasis; depending on vascular anatomy and procedural feasibility, particles or liquid embolic agents may be considered when more distal penetration or more comprehensive devascularisation is required [6].

We also clarified our follow‐up policy. While we provided a 1‐month follow‐up bronchoscopic image to document early regression and support effectiveness assessment, we consider routine scheduled bronchoscopy in asymptomatic patients to be potentially excessive. Instead, we recommend clinical follow‐up with bronchoscopy triggered by recurrent hemoptysis or blood‐tinged sputum (with contrast‐enhanced CT as an adjunct when appropriate, acknowledging its limited sensitivity for subtle endobronchial lesions).

The aetiology of bronchial Dieulafoy's disease is still unknown. Chronic inflammation of the respiratory tract is considered to play a role in the development of the disease, as a substantial proportion of the patients have a smoking history or a history of respiratory diseases including tuberculosis [4, 5]. However, it may also be congenital, as it can develop in young patients (in their 20s or younger) without a history of respiratory disease [3, 4, 5]. In the present case, the prior history of pneumonia may have contributed to the development of bronchial Dieulafoy's disease.

The strengths of this report include detailed evaluations before BAE: (i) bronchoscopy showing oozing bleeding from the polypoid lesion; (ii) convex‐probe EBUS demonstrating submucosal blood flow consistent with a calibre‐persistent artery; (iii) contrast‐enhanced CT showing lesion enhancement. Furthermore, lesion regression after BAE was confirmed by both bronchoscopy and contrast‐enhanced CT, which has rarely been reported elsewhere.

In conclusion, this case underscores the importance of a multimodal approach to the diagnosis of bronchial Dieulafoy's disease, particularly to obviate bronchial biopsy which can lead to fatal bleeding.

## Author Contributions

Masayoshi Higashiguchi and Yu Yamaguchi were the primary physicians of the patient. Masayoshi Higashiguchi and Satoshi Tetsumoto performed bronchoscopy. Yu Yamaguchi and Hideo Ishikawa performed bronchial artery embolization. Masayoshi Higashiguchi wrote the first draft of the manuscript. All authors read the manuscript and provided critical feedback. All authors approved submission of the final version of the manuscript.

## Funding

The authors have nothing to report.

## Consent

The authors declare that written informed consent was obtained for the publication of this manuscript and accompanying images using the consent form provided by the Journal.

## Conflicts of Interest

Hideo Ishikawa received lecture fees from Terumo Corporation, Boston Scientific Japan, Kaneka Corporation, and Asahi Intecc Co. Ltd.; and research funding from Terumo Corporation. Yu Yamaguchi received research funding from Terumo Corporation. The other authors declare no conflicts of interest.

## Data Availability

Data sharing not applicable to this article as no datasets were generated or analysed during the current study.
